# Primary atopic disorders: Monogenic insights into immunity

**DOI:** 10.70962/jhi.20260006

**Published:** 2026-05-07

**Authors:** Bhavi P. Modi, Cassandra McDonald, Liam Golding, Leandro B.R. Da Silva, Catherine M. Biggs, Julia Körholz, Elliot L. James, Stuart E. Turvey

**Affiliations:** 1 Department of Pediatrics, BC Children’s Hospital, University of British Columbia, Vancouver, Canada; 2 Department of Pediatrics, Faculty of Medicine and University Hospital Carl Gustav Carus, Technische Universität Dresden, Dresden, Germany; 3 German Center for Child and Adolescent Health, Partner site Leipzig/Dresden, Dresden, Germany

## Abstract

Primary atopic disorders (PADs), a subset of inborn errors of immunity, are a growing group of more than 50 monogenic diseases characterized by severe, early-onset allergic inflammation, often coexisting with immune deficiency or dysregulation. Once considered clinical curiosities, PADs have emerged as powerful human models for understanding immune homeostasis. Advances in next-generation sequencing have accelerated gene discovery, revealing that allergic disease can arise from disruption of interconnected regulatory systems rather than immune hyperactivity alone. Here, we synthesize insights from genetically defined PADs to illustrate how defects across epithelial barrier function, immune signaling, cytoskeletal organization, antigen receptor pathways, lymphocyte repertoire, and regulatory networks converge on impaired tolerance and persistent type 2 inflammation. Beyond mechanistic insights, PADs provide a framework for precision therapy enabling targeted use of cytokine inhibitors, JAK inhibitors, biologics, hematopoietic stem cell transplantation, and emerging gene therapies. These insights bridge rare and common disease, informing the pathophysiology and treatment of polygenic atopy.

## Teaser

Modi et al. review how primary atopic disorders, a group of monogenic inborn errors of immunity, reveal fundamental mechanisms regulating allergic inflammation. These human diseases show how disruptions in barrier integrity, immune signaling, cytoskeletal dynamics, and immune tolerance converge to drive severe type 2 inflammation and inform emerging precision therapies.

## Introduction

The global prevalence of atopic diseases, which span asthma, atopic dermatitis, food allergies, and allergic rhinitis, continues to increase, amplifying both health and economic burdens (1). Despite their ubiquity, the immunological mechanisms underlying these conditions remain incompletely understood, partly because common atopic diseases are shaped by complex polygenic architectures and environmental exposures. In contrast, a subset of individuals presents with early-onset, severe, and potentially life-threatening allergic diseases resulting from single-gene defects, collectively known as primary atopic disorders (PADs) (2, 3). PADs, or monogenic allergic diseases, are rare, clinically and genetically heterogeneous inborn errors of immunity (IEIs) caused by single-gene variants that affect immune function. Although rare, PADs provide genetically defined, causally anchored models of allergic inflammation and immune dysregulation that offer unique insights into core pathways shared with common atopic disease (4, 5).

To date, more than 50 distinct PAD-associated genes have been identified, involving cytokines, receptors, structural proteins, transcription factors, and signaling pathways. Notably, these genes span multiple International Union of Immunological Societies Expert Committee IEI classification categories, underscoring the biological diversity of PADs (see Table 1) (6).

**Table 1.** Distribution of PAD genes across the IUIS classification tables for IEIs

PAD gene	Inheritance	PAD mechanism
**Section I. Immunodeficiencies affecting cellular and humoral immunity**		
I-1. *T-B + SCID*		
*IL2RG*	XL, LOF	Abnormal T cell development and/or restriction of the TCR repertoire
*IL7R*	AR, LOF	Abnormal T cell development and/or restriction of the TCR repertoire
I-2. *T-B- SCID*		
*ADA*	AR, LOF	Abnormal T cell development and/or restriction of the TCR repertoire
*DCLRE1C*	AR, LOF	Abnormal T cell development and/or restriction of the TCR repertoire
*LIG4*	AR, LOF	Abnormal T cell development and/or restriction of the TCR repertoire
*RAG1*	AR, LOF	Abnormal T cell development and/or restriction of the TCR repertoire
*RAG2*	AR, LOF	Abnormal T cell development and/or restriction of the TCR repertoire
I-3. *CID, generally less profound than SCID*		
*DOCK8*	AR, LOF	Defects in actin cytoskeleton
*MSN*	XL, LOF	Defects in actin cytoskeleton
*STK4*	AR, LOF	Defects in actin cytoskeleton
*MALT1*	AR, LOF	Attenuated antigen receptor signaling, altered cellular metabolism
*RFXANK*	AR, LOF	Attenuated antigen receptor signaling
*ZAP70*	AR, LOF/GOF	Abnormal T cell development and/or restriction of the TCR repertoire
**Section II. CIDs with associated or syndromic features**		
II-1. *Immunodeficiency with congenital thrombocytopenia*		
*ARPC1B*	AR, LOF	Defects in actin cytoskeleton
*IKZF2*	AD, DN-GOF	Failure of immune tolerance
*WAS*	XL, LOF	Defects in actin cytoskeleton
*WIPF1*	AR, LOF	Defects in actin cytoskeleton
II-2. *DNA repair defects other than those listed in*Table 1		
No PADs		
II-3. *Thymic defects with additional congenital anomalies*		
*CHD7*	AD, LOF	Abnormal T cell development and/or restriction of the TCR repertoire
*TBX1*	AD	Abnormal T cell development and/or restriction of the TCR repertoire
22q11.2 deletion syndrome—large deletion (3 Mb) typically in chromosome 22 (TBX1)	AD	Abnormal T cell development and/or restriction of the TCR repertoire
II-4. *Immuno-osseous dysplasias*		
No PADs		
II-5. *Syndromes associated with elevated IgE and/or atopic disease not listed elsewhere (hyper-IgE syndromes* [7])		
*SPINK5*	AR, LOF	Skin barrier dysfunction
*CARD11*	AD, DN	Attenuated antigen receptor signaling, altered cellular metabolism
*ERBIN*	AD	Abnormal cytokine signaling
*IL6R*	AR, LOF	Abnormal cytokine signaling
*IL6ST*	AR, LOF	Abnormal cytokine signaling
*STAT3*	AD, DN-LOF	Abnormal cytokine signaling
*STAT6*	AD, GOF	Abnormal cytokine signaling
*TGFBR1*	AD	Abnormal cytokine signaling
*TGFBR2*	AD	Abnormal cytokine signaling
*ZNF341*	AR, LOF	Abnormal cytokine signaling
*PGM3*	AR, LOF	Altered cellular metabolism
II-6. *Defects of vitamin B12 and folate metabolism*		
No PADs		
II-7. *EDA-ID*		
No PADs		
II-8. *Calcium channel defects*		
No PADs		
II-9. *Other defects*		
*STAT5B*	AR, LOF and AD, DN-LOF	Abnormal cytokine signaling
**Section III. Predominantly antibody deficiencies**		
No PADs		
**Section IV. Diseases of immune dysregulation**		
IV-1. *FHL syndromes*		
No PADs		
IV-2. *FHL syndromes with hypopigmentation*		
No PADs		
IV-3. *Tregdefects*		
*FOXP3*	XL, LOF	Failure of immune tolerance
*CTLA4*	AD, haploinsufficiency	Failure of immune tolerance
*IKZF1*	AD, GOF	Abnormal T cell development and/or restriction of the TCR repertoire
*IL2RA*	AR, LOF	Failure of immune tolerance
*IL2RB*	AR, LOF	Failure of immune tolerance
*STAT3*	AD, GOF	Abnormal cytokine signaling
IV-4. *Autoimmunity with or without lymphoproliferation*		
*NCKAP1L*	AR, LOF	Defects in actin cytoskeleton
*JAK1*	AD, GOF	Abnormal cytokine signaling
*SOCS1*	AD, LOF	Abnormal cytokine signaling
IV-5. *Immune dysregulation with colitis*		
*RHBDF2*	AR, LOF	Skin barrier dysfunction
IV-6. *ALPS* (*Canale–Smith syndrome*)		
No PADs		
IV-7. *Susceptibility to EBV and lymphoproliferative conditions*		
*CARMIL2*	AR, LOF	Attenuated antigen receptor signaling
**Section V. Congenital defects of phagocyte number or function**		
No PADs		
**Section VI. Defects in intrinsic and innate immunity**		
VI-1. *MSMD*		
*TBX21*	AR, LOF	Abnormal cytokine signaling, abnormal T cell development, and/or restriction of the TCR repertoire
*TYK2*	AR, LOF	Abnormal cytokine signaling
VI-2. *Epidermodysplasia verruciformis (HPV)*		
No PADs		
VI-3. *Predisposition to severe viral infection*		
No PADs		
VI-4. *HSE*		
No PADs		
VI-5. *Predisposition to invasive fungal diseases*		
No PADs		
VI-6. *Predisposition to mucocutaneous candidiasis*		
*STAT1*	AD, GOF	Abnormal cytokine signaling
VI-7. *TLR signaling pathway deficiency*		
No PADs		
VI-8. *Other IEIs related to nonhematopoietic tissues*		
No PADs		
VI-9. *Other IEIs related to leukocytes*		
No PADs		
**Section VII. Autoinflammatory disorders**		
VII-1. *Type 1 interferonopathies*		
No PADs		
VII-2. *Defects affecting the inflammasome*		
*PLCG2*	AD, GOF/LOF	Abnormal immune signaling
VII-3. *Non–inflammasome-related conditions*		
*LYN*	AD, GOF	Abnormal immune signaling
**Section VIII. Complement deficiencies**		
No PADs		
**Section IX. Bone marrow failure**		
No PADs		
**Section X. Phenocopies of IEIs associated with autoantibodies or somatic variants**		
X-1. *Associated with somatic variants*		
*STAT5B*	Somatic, GOF	Abnormal cytokine signaling
**Genes not included in the IUIS 2024-update table**		
*CARD14*	AD, LOF	Attenuated antigen receptor signaling
*CDSN*	AR, LOF	Skin barrier dysfunction
*DSG1*	AR, LOF	Skin barrier dysfunction
*DSP*	AD, LOF	Skin barrier dysfunction
*FLG*	AD or AR, LOF	Skin barrier dysfunction
*OSMR*	AR, LOF	Abnormal cytokine signaling
*ZBTB7B*	AD	Abnormal T cell development and/or restriction of the TCR repertoire

PAD genes are shown within the IUIS classification of IEIs in which disorders are currently categorized into 10 tables, with subtables segregating groups of disorders into overlapping phenotypes (2024 update) (6). For each gene, the mode of inheritance and the predominant pathogenic mechanism contributing to atopic disease are indicated. IUIS disease categories in which no PAD genes have been identified are also shown and noted accordingly. In addition, genes known to cause monogenic atopic diseases, but not yet identified within the IUIS framework, are also listed. IUIS, International Union of Immunological Societies; HPV, human papilloma virus; SCID, severe combined immunodeficiency; CID, combined immunodeficiency; EDA-ID, anhidrotic ectodermal dysplasia with immunodeficiency; FHL, familial hemophagocytic lymphohistiocytosis; ALPS, autoimmune lymphoproliferative syndrome; MSMD, Mendelian susceptibility to mycobacterial disease; HSE, herpes simplex encephalitis; AD, autosomal dominant; AR, autosomal recessive; DN, dominant negative; XL, X-linked; LOF, loss-of-function; GOF, gain-of-function.

Importantly, while PADs are monogenic by definition, their clinical manifestations are often modulated by environmental exposures, modifier genes, and epigenetic influences, highlighting the complexity and plasticity of human immune responses (8). Clinical manifestations between patients can be heterogeneous, with atopic features variably present and not always clinically dominant, reflecting underlying differences in pathway involvement. The distinction between monogenic and complex disease is increasingly blurred, as rare variants in PAD-associated genes are now being identified in subsets of patients with common atopic conditions (9). In parallel, genome-wide association studies of polygenic atopic diseases have repeatedly implicated noncoding regulatory variants that modulate the expression of PAD-associated genes, thereby increasing disease susceptibility (10, 11). Taken together, these findings support a model in which monogenic and polygenic atopic diseases exist along a continuum of shared biological mechanisms rather than as categorically distinct entities (12).

Studying human PADs has provided powerful mechanistic insights into type 2 immunity, skin–immune crosstalk, and the regulation of immunological tolerance. As these pathways are also central to more common allergic diseases, the deeper understanding facilitated by the study of monogenic PADs has accelerated biomarker discovery and the identification of treatable pathways (4). One example is the use of biologics such as dupilumab, originally approved for atopic dermatitis, now being repurposed for rare IL-4/IL-13 pathway-driven monogenic disorders including *STAT6* gain-of-function (GOF) disease and other disorders characterized by severe type 2 skewed inflammation such as those caused by damaging variants in *STAT3*, *CARD11*, and *DOCK8* (13, 14, 15, 16).

This review highlights how the study of monogenic PADs has advanced our understanding of fundamental immunobiology, clarified mechanisms of barrier dysfunction and type 2 inflammation, and informed new therapeutic opportunities through pathway-specific targeting. Rather than an exhaustive mechanistic review of all PADs, we focus on a curated group of well-characterized disorders that illustrate key biological principles. Through this lens, we highlight how insights from rare monogenic disease are reshaping precision approaches in allergy and immunology, with relevance extending beyond affected individuals to the broader population living with allergic and inflammatory disorders. Distinct from prior reviews that have typically catalogued the expanding spectrum of PAD genes, this review adopts a unifying, pathway-centered framework that emphasizes mechanistic convergence to demonstrate how genetically defined disorders illuminate shared pathways underlying common allergic disease and inform scalable precision strategies beyond rare disease.

## Real-world lessons in human immunobiology

**Figure 1.**
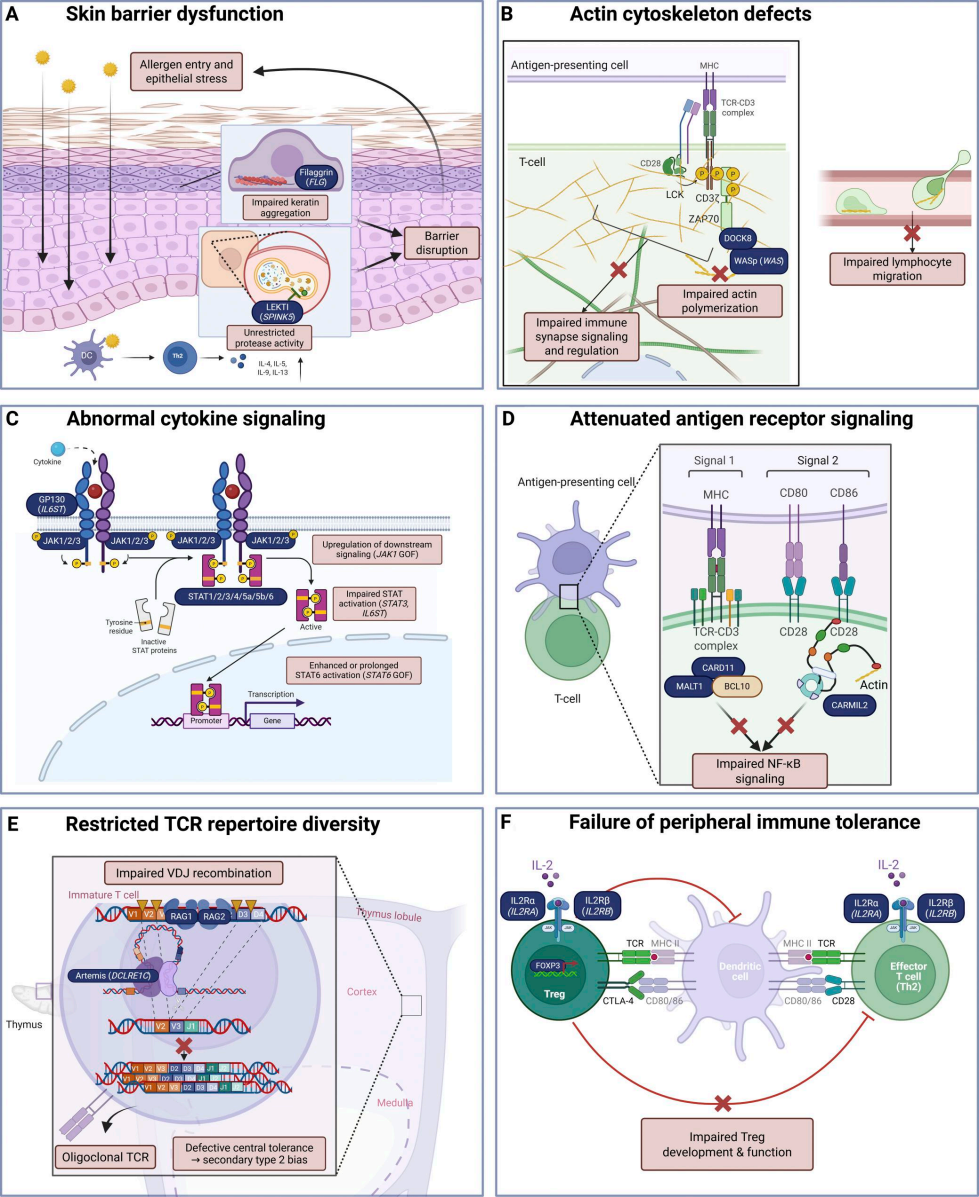
**Mechanistic pathways contributing to PADs.** PADs result from heterogeneous molecular defects that converge on exaggerated type 2 immune responses and impaired immune regulation. **(A–F)** Shown are representative mechanisms including skin barrier dysfunction (A), actin cytoskeleton defects affecting immune synapse formation and cell migration (B), abnormal cytokine signaling with amplified JAK-STAT activity (C), attenuated antigen receptor and costimulatory signaling (D), restricted TCR repertoire diversity (E), and failure of peripheral immune tolerance due to impaired Treg function (F). The figure was created in BioRender.

Monogenic allergic disorders are powerful human models for mapping pathways that underlie common complex allergic diseases. Unlike common allergic diseases, PADs enable direct genotype–phenotype correlations, allowing causal relationships between genetic defects and clinical outcomes to be defined. The following examples illustrate how PADs have clarified fundamental mechanisms governing epithelial barrier integrity, type 2 immunity, immune cell development, and immune tolerance to environmental and self-antigens (see Fig. 1).

### Skin barrier dysfunction: *SPINK5* and *FLG*

A strong skin barrier is essential for preventing allergies because it limits the entry of environmental allergens and microbes, reducing immune activation and lowering the risk of sensitization. PADs such as Netherton syndrome (OMIM 256500) and ichthyosis vulgaris (OMIM 146700), caused by damaging variants in the *SPINK5* and *FLG* genes, respectively, have provided direct human evidence for the critical role of the skin barrier in preventing allergic sensitization.

*SPINK5* encodes lymphoepithelial Kazal-type–related inhibitor (LEKTI), a serine protease inhibitor expressed by keratinocytes that is essential for normal skin barrier integrity. Normal LEKTI activity is important for the suppression of stratum corneum serine protease activity, which prevents excessive peeling of the stratum corneum (see Fig. 1 A) (17). Individuals with biallelic loss-of-function (LOF) variants in *SPINK5* develop Netherton syndrome, which is clinically characterized by congenital erythroderma, early-onset atopic dermatitis, elevated IgE levels, and hypereosinophilia combined with the distinctive “bamboo” hair shaft abnormality of trichorrhexis invaginata (18, 19). The loss of functional LEKTI results in unregulated protease activity that damages the skin barrier and triggers inflammation (20). Similarly, LOF variants in *FLG*, which encodes filaggrin (filament-aggregating protein), compromise the integrity of the skin barrier (21, 22). Filaggrin is a key structural protein that aggregates keratin filaments during terminal keratinocyte differentiation and is subsequently degraded into natural moisturizing factors essential for hydration and maintenance of the stratum corneum (see Fig. 1 A) (23). *FLG* variants act in a semidominant manner, meaning that individuals with monoallelic pathogenic variants in *FLG* usually have mild ichthyosis vulgaris or more subtle signs such as dry skin or hyperlinear palms, while those with biallelic pathogenic variants develop more severe skin disease (21, 24). Furthermore, hypomorphic variants in *FLG* have been implicated in the progression from atopic dermatitis to allergic airway diseases (25, 26).

Together, these discoveries have reframed epithelial barriers as active regulators of immune homeostasis, establishing barrier integrity as a critical upstream determinant of allergic sensitization and ensuing downstream type 2 immune skewing.

### Defects in the actin cytoskeleton: *WAS*, *DOCK8*

The actin cytoskeleton is essential for normal lymphocyte function, and PADs affecting actin regulation have provided important mechanistic insights into the cellular origins of allergic inflammation. Dynamic actin remodeling supports key T cell processes, including immunological synapse formation, T cell receptor (TCR) signaling, stable cell–cell interactions with antigen-presenting cells, and effective activation. Regulatory T cells (Tregs) likewise depend on intact actin remodeling for migration, suppressive function, and the maintenance of peripheral tolerance for controlling inflammatory T cell responses such as T helper 2 cell (Th2)–type inflammation (27). Consequently, LOF variants in genes that regulate actin polymerization, such as *DOCK8*, *WAS*, *NCKAP1L*, *WIPF1*, *ARPC1B*, *STK4*, and *MSN*, characteristically lead to a phenotype that includes severe eczema, recurrent infections, allergic disease, autoimmunity, and exaggerated type 2 inflammation (28, 29, 30, 31, 32, 33).

Although the precise mechanisms may vary across individual genes, several convergent immunological themes have emerged. For example, patients with biallelic LOF *DOCK8* variants present with autosomal recessive (AR) hyper-IgE syndrome (OMIM 243700), a severe disorder characterized by combined immunodeficiency, marked atopic dermatitis and allergies, extensive cutaneous viral infections, increased cancer susceptibility, and very high serum IgE levels (34, 35). Mechanistic studies show that DOCK8 deficiency impairs cytoskeletal reorganization required for lymphocyte migration, immune synapse formation, and survival (see Fig. 1 B) (36), biasing CD4^+^ T cells to a Th2 effector fate (37) and impairing B cell signaling and metabolism (38). DOCK8 also interacts with STAT3 to restrain IL-13–producing T cells (39). As a result, *DOCK8* LOF variants impair the ability of T and B cells to migrate to sites of infection or to secondary lymphoid organs, compromise germinal center formation, and remove critical regulatory mechanisms that normally limit excessive Th2 responses. A related PAD, Wiskott–Aldrich syndrome (WAS) (OMIM 301000), is an X-linked recessive condition caused by pathogenic variants in the *WAS* gene encoding the actin-regulatory protein WASp, a hematopoietic-specific actin-regulatory protein that orchestrates actin polymerization and cytoskeletal remodeling in immune cells (40). Clinically, WAS is defined by the classic triad of eczema, thrombocytopenia with small platelets, and immunodeficiency (41, 42, 43). Mechanistically, individuals with WAS exhibit defective immune synapse formation, impaired T cell activation, and abnormal Treg function, all of which contribute to elevated IgE, food allergy, and broader immune dysregulation, further illustrating the link between cytoskeletal dysfunction and allergic disease (44, 45, 46) (see Fig. 1 B).

Collectively, these PADs demonstrate that intact actin dynamics are essential for effective immune signaling and regulation, and that disruption preferentially weakens tolerance and promotes type 2–skewed inflammation rather than global immune failure.

### Abnormal cytokine signaling: *JAK1*, *STAT6*, *STAT3*

Cytokine signaling is central to immune cell activation, coordinating how lymphoid, myeloid, and epithelial cells interpret environmental cues and shaping the magnitude and quality of immune responses. PADs provide compelling evidence from the intact human system that exaggerated type 2 immune skewing leads directly to severe allergic inflammation. The key pro-allergic cytokines IL-4 and IL-13 signal through receptors that activate the kinase JAK1, which then phosphorylates and activates the transcription factor STAT6 (47). This cytokine–JAK1–STAT6 axis is central to Th2 polarization and B cell class switching to IgE (see Fig. 1 C). Persistent activation of this pathway amplifies eosinophilic inflammation, tissue remodeling in the skin and airways, epithelial barrier dysfunction, and susceptibility to multisystem allergic disease; ultimately driving the clinical manifestations of atopic dermatitis, asthma, food allergy, and allergic rhinitis (48, 49).

GOF variants in both *JAK1* and *STAT6* lead to Th2 skewing and a range of allergic manifestations. Germline heterozygous *JAK1* GOF variants were first linked to severe allergic inflammation in 2017 in a multigenerational family with multiple affected members (50) (OMIM 618999), and additional cases have since expanded the phenotypic spectrum (9, 51, 52, 53). The importance of the JAK1-STAT6 signaling axis was further underscored by the recent discovery of heterozygous *STAT6* GOF variants, as reported in 2023 in a cohort of 16 individuals presenting with childhood-onset allergic immune dysregulation, including widespread atopic dermatitis, gastrointestinal disease, asthma, IgE-mediated food allergy, and anaphylaxis, inherited in an autosomal dominant (AD) pattern (13) (OMIM 620532). A phenotype that has now been replicated across multiple independent reports of AD *STAT6* GOF disease (13, 54, 55, 56, 57, 58, 59). Beyond Th2 skewing, excessive JAK1-STAT6 signaling may also impair immune tolerance by functionally destabilizing Tregs. Heightened IL-4/IL-13 signaling has been associated with repression of FOXP3 transcription and reduced Treg suppression, thereby contributing to allergic inflammation not only through effector expansion but also through impaired regulation (60, 61). Together, these discoveries firmly establish the JAK1-STAT6 pathway as a critical driver of monogenic allergic disease and highlight how dysregulated type 2 cytokine signaling can produce severe, early-onset atopic inflammation in humans.

In contrast to disorders caused by intrinsic amplification of type 2 cytokine signaling, STAT3-related PADs exemplify how loss of immune counter-regulatory and barrier-protective programs permits secondary dominance of allergic inflammation. Arguably, the best-known PAD of all is AD hyper-IgE syndrome (AD-HIES) (OMIM 147060) or Job’s syndrome (62), which in 2007 was found to be caused by heterozygous *STAT3* variants (63, 64). Missense and short in-frame deletions concentrated in the SH2 and DNA-binding domains of STAT3 are the major cause of AD-HIES, and disease-causing *STAT3* variants appear to act through dominant-negative mechanisms rather than haploinsufficiency (65). *STAT3* dominant-negative variants cause disease because the abnormal protein interferes with the normal allele, leading to insufficient STAT3 activity across critical cytokine pathways, resulting in defective Th17 immunity, impaired antibody responses, and skewing toward Th2 phenotypes (66, 67, 68, 69, 70). Patients with AD-HIES typically present with immunodeficiency impacting mucosal immunity with particular susceptibility to fungal and extracellular bacterial infections, as well as high IgE, atopic dermatitis, and abnormalities of bone and connective tissue (66, 71). Further highlighting the importance of STAT3 signaling in restraining allergic disease, multiple PAD-associated genes disrupt pathways that converge on STAT3 activation and STAT3-dependent immune programs. For example, variants in *IL6ST* and *IL6R* impair IL-6 family cytokine signaling upstream of STAT3, whereas *ZNF341* deficiency reduces STAT3 transcription itself. Similarly, *IL21R* defects disrupt IL-21–mediated STAT3 activation, and cytoskeletal defects such as *DOCK8* deficiency impair immune synapse formation and downstream signaling required for optimal STAT3 pathway activation. Collectively, these defects attenuate STAT3-driven immune programs, resulting in impaired Th17 differentiation, defective regulatory cytokine signaling, and a shift toward unchecked type 2 inflammation (72, 73, 74, 75, 76, 77, 78).

Together, disorders affecting *STAT6*, *JAK1*, and *STAT3* illustrate how perturbations in key cytokine signaling pathways can shift the immune system toward unchecked type 2 inflammation, revealing the fundamental centrality of these nodes in maintaining immune balance and preventing allergic disease.

### Attenuated antigen receptor signaling: *CARD11*, *MALT1*, *CARMIL2*

Antigen receptor signal strength is a key determinant of how immune cells interpret antigen exposure and play a major role in allergic disease. For example, in T cells, weaker or improperly regulated TCR signals favor Th2 differentiation, whereas stronger and more sustained signaling promotes Th1 or Th17 responses and effective immune regulation (79, 80). While defects in actin cytoskeletal dynamics (as outlined above) can skew toward pro-allergic responses by weakening effective antigen receptor signaling, primary defects in antigen receptor signal transduction can also directly reduce signal strength favoring Th2 immune responses. At the molecular level, antigen receptor signal strength is encoded by proximal signaling complexes that link receptor engagement to transcriptional programs, among which the CARD11–BCL10–MALT1 (CBM) complex plays a central role.

The CBM complex couples TCR and B cell receptor engagement to downstream NF-κB (and related) signaling programs (see Fig. 1 D). Germline disorders affecting this pathway are often referred to collectively as CBM-opathies (81). Complete (biallelic, LOF) deficiency of any of the three CBM components (*CARD11*, *BCL10*, or *MALT1*) typically causes a severe, overlapping phenotype dominated by combined immunodeficiency and immune dysregulation; in contrast, defects that attenuate CBM function without abolishing it can present with prominent allergic disease. Heterozygous dominant-negative (dominant-interfering) *CARD11* variants cause “CARD11-associated atopy with dominant interference of NF-κB signaling” (CADINS; OMIM 617638) (82, 83, 84). The dominant-negative interference of the defective CARD11 leads to inadequate CBM complex assembly and impaired NF-κB and mTORC1 signal transduction. In addition to severe atopy, the CADINS phenotype is now recognized to encompass frequent sinopulmonary infections, cutaneous viral infections, neutropenia, hypogammaglobulinemia, and lymphoma (85). Similarly, biallelic hypomorphic *MALT1* variants that preserve partial CBM signaling have been associated with prominent immune dysregulation and allergic manifestations, including eczema and elevated IgE, phenocopying key aspects of *CARD11*-associated atopic disease (86, 87) (OMIM 615468). To date, humans with hypomorphic partial loss of BCL10 function have not yet been reported.

Reinforcing the paradigm that intact antigen receptor signaling is required to prevent persistent skewing toward type 2 immunity, human CARMIL2 deficiency (OMIM 618131) causes a PAD-like phenotype in which impaired CD28-dependent costimulatory signaling (i.e., signal two) leads to chronic allergic inflammation (see Fig. 1 D). *CARMIL2* (also known as *RLTPR*) encodes a cytosolic protein that is essential for CD28 costimulation in T cells. Biallelic LOF variants in *CARMIL2* impair CD28-dependent T cell costimulatory signaling, resulting in defective downstream NF-κB activation, attenuation of B cell receptor–induced NF-κB signaling in B cells, and reduced circulating Treg numbers (88, 89, 90). Clinically, affected individuals typically present with early-onset atopic dermatitis, elevated IgE, food allergy, and asthma, alongside recurrent respiratory and cutaneous infections, particularly with viral pathogens such as Epstein-Barr virus (EBV) (including EBV^+^ smooth muscle tumors), human papillomavirus, molluscum contagiosum, and herpesviruses, as well as recurrent sinopulmonary bacterial infections. Inflammatory gastrointestinal disease resembling inflammatory bowel disease is also common. In contrast, complete AR CD28 deficiency in humans appears to produce a much narrower phenotype characterized primarily by susceptibility to cutaneous human papilloma virus infection rather than the broad allergic and inflammatory manifestations seen in CARMIL2 deficiency, suggesting that CARMIL2 has nonredundant roles beyond canonical CD28 signaling (90, 91). Collectively, these observations highlight how disruption of CD28-associated signaling pathways can differentially perturb immune regulation and promote allergic inflammation (88, 89, 90, 92, 93).

When considered as a group, these disorders illustrate that graded reductions in antigen receptor and costimulatory signal strength preferentially impair immune regulation while preserving lymphocyte survival, thereby biasing the immune system toward persistent type 2 inflammation. CBM-opathies and related costimulatory defects highlight antigen receptor signal tuning as a central mechanism underlying PADs.

### Abnormal T cell development and/or restriction of the TCR repertoire: *RAG1*, *RAG2*, and other genetic causes of Omenn syndrome

TCR repertoire diversity is fundamental to immune health, enabling broad antigen recognition, effective immune regulation, and protection from both infection and pathological immune skewing. Omenn syndrome illustrates in humans how profound TCR repertoire constriction can be coupled to severe type 2–skewed inflammation (OMIM 603554). Omenn syndrome is characterized by a markedly restricted, oligoclonal T cell repertoire with activated T cells and prominent Th2-associated features (see Fig. 1 E). In addition to susceptibility to severe infections, these T cells drive erythroderma, lymphadenopathy, hepatosplenomegaly, eosinophilia, very high serum IgE, failure to thrive, and life-threatening immune dysregulation. The syndrome was first described by Omenn in 1965 (94). Most commonly, Omenn syndrome occurs in the setting of leaky/atypical SCID due to biallelic hypomorphic variants in *RAG1* or *RAG2* that impair, but do not abolish, V(D)J recombination and thereby generate a severely restricted TCR repertoire (95, 96). However, an Omenn phenotype can also arise from variants in other SCID-related genes that partially impair T cell development or antigen receptor generation; examples include *DCLRE1C* (Artemis), *LIG4*, *RMRP*, *ADA*, and 22q11.2 deletion syndrome.

Collectively, Omenn syndrome and related “leaky SCID” conditions demonstrate that profound restriction of TCR diversity is sufficient to drive severe immune dysregulation with prominent type 2 inflammation, underscoring repertoire diversity as a central safeguard against allergic disease.

### Failure of immune tolerance: *FOXP3*, *IL2RA*, *IL2RB*

Immune tolerance failure in PADs reflects defects in the mechanisms that normally prevent inappropriate immune activation against harmless antigens, such as food proteins, commensal microbes, and environmental allergens. Central tolerance operates during lymphocyte development in the thymus and bone marrow, where self-reactive or poorly regulated clones are deleted or edited. PADs affecting these processes lead to the emergence of oligoclonal, poorly regulated lymphocytes that are prone to type 2 skewing. Such failures of central tolerance are described above in the section on “Restricted TCR repertoire diversity.” Peripheral tolerance restrains immune responses after lymphocytes exit primary lymphoid organs and depends on Tregs, as well as other factors described previously including costimulation, antigen receptor signal tuning, cytokine balance, and tissue barriers (see Fig. 1 F). The unifying concept is that human PADs highlight that both central and peripheral tolerance are actively maintained processes, and genetic disruption at either level can lower the threshold for type 2 immune activation and allergic disease.

Tregs prevent allergic disease by actively suppressing type 2 effector responses and maintaining immune tolerance to environmental and dietary antigens at barrier tissues (see Fig. 1 F). Tregs play a central role in regulating peripheral tolerance. This is exemplified by damaging variants in the Treg fate-determining transcription factor FOXP3, which is essential for the transcriptional programs that enforce self-tolerance and prevent autoimmunity. The absence of fully functional FOXP3 causes immune dysregulation, polyendocrinopathy, enteropathy, X-linked (IPEX) syndrome (97, 98, 99). IPEX syndrome has X-linked inheritance and presents with profound, early-onset immune dysregulation, typically manifesting in infancy with severe, treatment-refractory atopic dermatitis, multiple IgE-mediated food allergies, and chronic autoimmune enteropathy leading to diarrhea, malabsorption, and failure to thrive. Affected individuals frequently develop endocrinopathies such as type 1 diabetes mellitus and autoimmune thyroid disease. They also show markedly elevated IgE levels, eosinophilia, and a broad spectrum of autoimmune manifestations that can involve the skin, gut, pancreas, thyroid, kidneys, and hematologic system, often resulting in life-threatening disease without definitive immune-directed therapy such as hematopoietic stem cell transplantation (HSCT) (100, 101).

Following this biological theme, biallelic LOF variants in *IL2RA* (OMIM 606367) and *IL2RB* (OMIM 618495), which encode the α and β subunits of the interleukin-2 receptor, impair high-affinity IL-2 signaling required for Treg survival, proliferation, and function. As a consequence, these defects result in AR IPEX-like immune dysregulation characterized by early-onset eczema, food allergy, elevated IgE, autoimmunity, and susceptibility to infection (102, 103, 104, 105).

Collectively, these observations establish Treg dysfunction as a unifying feature across many PADs arising from diverse mechanisms, including impaired antigen receptor signaling (*CARD11*, *MALT1*, *CARMIL2*), cytoskeletal defects (*WAS*, *DOCK8*, *ARPC1B*), and excessive type 2 cytokine signaling (*JAK1*, *STAT6*). These defects impair Treg development, survival, or suppressive capacity, underscoring the central role of intact Treg biology in maintaining immune tolerance and protecting against allergic disease.

In summary, studying PADs has revealed that allergic disease frequently emerges from imbalances in immune signaling and not complete immune failure. Disruption of barrier integrity, cytoskeletal dynamics, cytokine pathways, antigen receptor signal strength, repertoire diversity, or regulatory circuits all converge on a common outcome—reduced immune tolerance and persistent type 2 inflammation. PADs therefore provide a unifying framework for understanding how immune balance is actively maintained and how perturbations drive allergic disease. These mechanistic insights provide a direct rationale for pathway-targeted therapies in PADs and inform precision approaches to common allergic disease.

## Translating genetic insights into targeted therapy for people living with PADs

In addition to advancing our understanding of immune dysregulation, the study of monogenic PADs has also opened the door to targeted, mechanism-based therapies. In contrast to polygenic allergic disease, where treatment decisions are largely symptom-based, monogenic disorders implicate individual immune pathways and biomarkers that can be modulated with new or repurposed drugs. Therefore, PADs present a unique opportunity for precision medicine where the causal variant identifies the pathway, and the pathway identifies the therapeutic target. This gene–pathway–therapy framework has enabled mechanism-guided treatment for a growing number of individuals with PADs and has informed therapies ranging from targeted pathway modulation to immune system replacement (see Table 2).

**Table 2.** Targeted and disease-modifying therapies reported in PADs

Therapy	Mechanism	PAD mechanism targeted	Typical clinical indication	Utilized in PADs	Select references
Dupilumab	Anti-IL-4Rα mAb blocking IL-4/IL-13 signaling	Type 2 cytokine amplification	Atopic dermatitis; asthma; chronic rhinosinusitis with nasal polyps; eosinophilic esophagitis	*STAT3* DN; *STAT6* GOF; *DOCK8* deficiency (HIES); *CARD11* (CADINS); IPEX; *ZNF341* deficiency; Omenn syndrome; Netherton syndrome	(13, 58, 59, 106, 107, 108, 109, 110, 111, 112, 113, 114)
JAKinibs (e.g., baricitinib, tofacitinib, ruxolitinib, upadacitinib)	JAK-STAT pathway inhibition	Cytokine signaling amplification or loss of counter-regulation	Inflammatory diseases (agent-specific); atopic dermatitis for upadacitinib (systemic) and ruxolitinib cream (topical)	*STAT6* GOF; *JAK1* GOF; selected PADs with JAK-STAT dysregulation	(9, 13, 50, 51, 53, 57, 115)
Omalizumab	Anti-IgE mAb	Effector cell (IgE-FcεRI) pathway	Allergic asthma; chronic spontaneous urticaria; chronic rhinosinusitis with nasal polyps; IgE-mediated food allergy (reduction of reactions with accidental exposure)	Selected IgE- or urticaria-predominant PAD phenotypes	(116, 117, 118, 119)
Mepolizumab, benralizumab, reslizumab	Anti-IL-5 (mepolizumab, reslizumab) or anti-IL-5Rα (benralizumab) mAbs; eosinophil depletion	Eosinophil-dominant type 2 inflammation	Severe eosinophilic asthma (all); eosinophilic granulomatosis with polyangiitis (mepolizumab)	PADs with prominent eosinophilia or eosinophilic asthma (limited to several case reports)	(59, 120)
HSCT	Replacement of defective hematopoietic and blood-derived immune compartments	Global immune dysregulation/tolerance failure	Curative therapy for selected IEIs	WAS; *DOCK8* deficiency; *STAT3* DN (selected); *CARMIL2* deficiency; Omenn syndrome; *IL2RA*/*IL2RB* deficiency	(7, 121, 122, 123, 124, 125, 126)

Summary of immunomodulatory biologics, small-molecule inhibitors, and HSCT that have been used to treat PADs, organized by therapeutic mechanism, the pathogenic pathway targeted, and reported clinical indications. Reported PAD use is derived predominantly from case reports and small case series rather than randomized controlled trials. Therapeutic efficacy appears greatest when the drug’s mechanism of action aligns closely with the underlying molecular and immunological pathogenesis of the specific PAD.

Because many PADs affect immune pathways that are already well characterized in other disease contexts, they are particularly amenable to therapeutic repurposing (127). Some of the most significant clinical advances in PADs have come from adapting therapies originally developed for other immune-mediated or neoplastic diseases. Clinicians have successfully applied existing drugs, including JAK inhibitors (JAKinibs) and anticytokine biologics, soon after molecular diagnosis, drastically improving outcomes in diseases that were previously difficult or impossible to treat. Repurposing also lowers barriers to clinical use as safety profiles and pharmacokinetics are already well established. In this section, we review recent successes and explore emerging directions in immune pathway-targeting therapeutics. While these treatment approaches for PADs are grounded in the mechanistic understanding of the underlying pathways, it should be noted that these uses are largely off-label and based on case reports or small series becasue classic clinical trials are typically not feasible in these rare diseases.

### JAK inhibitors

An example of successful and rapid repurposing is the treatment of JAK1 GOF, first described in 2017, which causes severe atopy, failure to thrive, and eosinophilic infiltration across multiple organs (50). At the time of its discovery, JAKinibs such as ruxolitinib were already in clinical use for myeloproliferative disorders. When the first individuals with JAK1 GOF were identified and the variant was characterized, ruxolitinib was applied to primary cells in vitro, resulting in decreased activation of JAK1, and providing evidence supporting the clinical application of the medication. Subsequently, a growing number of JAK1 GOF patients were treated with oral JAKinibs resulting in clinical resolution of the majority of symptoms, as well as improved growth (9, 50, 51, 53, 115). JAKinibs have also been remarkably effective in STAT6 GOF disorders, where they disrupt the feedforward cytokine signaling loop in which activated STAT6 amplifies IL-4– and IL-13–driven type 2 gene expression (13, 57).

Current evidence suggests that JAKinibs are most effective in PADs driven by cytokine hyperactivation, particularly JAK1-STAT6–mediated type 2 signaling, but have limited or adjunctive roles in PADs caused by structural immune defects or failures of immune tolerance, where curative strategies such as HSCT remain central (see Table 2).

### IL-4/IL-13 blockade with dupilumab

Other impactful therapeutic advances in PADs have come from targeting cytokine signaling central to the Th2 immune response. The IL-4/IL-13 signaling axis, mediated through the shared IL-4 receptor α (IL-4Rα) subunit and the transcription factor STAT6, lies at the heart of pro-allergic inflammation (49). Dupilumab is a fully human monoclonal antibody targeting IL-4Rα, thereby inhibiting both IL-4 and IL-13 signaling and effectively suppressing type 2 inflammation (128).

The discovery and subsequent treatment of the first patients with *STAT6* GOF variants in 2023 provided a compelling example of targeting IL-4Rα with dupilumab (14). STAT6 GOF is characterized by early-onset severe allergic inflammation, and molecular analysis revealed excessive transcriptional responses to IL-4 and IL-13 stimulation compared with healthy controls, consistent with enhanced Th2 polarization. These patients were often refractory to conventional therapies, but several patients have responded remarkably well to dupilumab with notable improvement in skin condition, growth parameters, and overall health (13, 58, 59). Today in the absence of direct comparative studies, the choice between JAK inhibition and IL-4Rα blockade in *STAT6* GOF disease has largely been guided by local drug availability, regulatory approval, and individual patient considerations, highlighting the need for prospective studies to inform optimal therapeutic selection.

In Netherton syndrome caused by biallelic LOF variants in *SPINK5*, dupilumab has repeatedly been shown in case reports and small series to improve severe atopic dermatitis, pruritus, erythroderma, and quality of life (106, 107, 108, 109, 110). The therapeutic rationale is that loss of LEKTI leads to barrier breakdown and protease-driven inflammation that secondarily activates IL-4/IL-13–mediated type 2 immune responses; dupilumab interrupts this downstream inflammatory axis without worsening infection risk. Recent work suggests that dupilumab may also induce TIM-3 expression and suppress myeloid dendritic cell function, indicating that mechanisms beyond direct blockade of type 2 cytokine signaling may contribute to its therapeutic effects (129). Overall, dupilumab has emerged as one of the most consistently effective targeted therapies reported for Netherton syndrome, despite the primary defect being epithelial rather than immune.

Other PADs with overlapping Th2 phenotypes have similarly responded to anti-IL-4Rα therapy; for example, patients with severe atopy caused by variants in *CARD11*, *DOCK8*, *STAT3*, and *ZNF341* have shown clinical improvement following dupilumab treatment (see Table 2) (16, 111, 112, 113, 114). Although it should be recognized that in these conditions dupilumab served as targeted anti-inflammatory therapy rather than disease-correcting treatment, these cases highlight that IL-4Rα blockade may be beneficial across a range of PADs with Th2-skewed inflammation, regardless of the causative genetic defect. Together, these observations establish excessive IL-4/IL-13 signaling as a convergent, therapeutically tractable feature across genetically diverse PADs.

### Omalizumab to bind free IgE

Omalizumab is a humanized monoclonal antibody that sequesters circulating IgE, thereby reducing FcεRI engagement on mast cells and basophils. In PADs, omalizumab has been used primarily as a symptomatic, downstream therapy to mitigate IgE-mediated manifestations rather than to correct the underlying immunological defect. Omalizumab has been reported to offer some benefit in patients with PADs caused by genetic changes in *STAT3* (116, 117), *WAS* (118), *DOCK8* (130), and *CARD11* (119), particularly in patients with prominent allergic asthma, urticaria, or anaphylaxis (see Table 2). Consistent with its mechanism of action, omalizumab does not modify upstream immune programming and is best viewed as an adjunctive therapy for IgE-driven symptoms in selected PAD phenotypes.

### Anti-IL-5/IL-5Rα monoclonal antibodies to reduce eosinophil survival

Anti-IL-5/IL-5Rα monoclonal antibodies reduce eosinophilic inflammation, a key (but single) effector arm of type 2 immunity. Mepolizumab and reslizumab bind IL-5, inhibiting eosinophil maturation, activation, and survival, whereas benralizumab binds IL-5Rα on eosinophils and basophils, inducing antibody-dependent cell-mediated cytotoxicity and eosinophil depletion. In PADs, these agents have been used occasionally in a phenotype-directed, adjunctive manner for eosinophil-dominant disease, particularly severe eosinophilic asthma or hypereosinophilic complications. Consistent with their downstream mechanism of action, anti-IL-5/IL-5Rα therapies are generally insufficient as monotherapy for broader atopic manifestations in PADs and are best reserved for cases in which eosinophilia is a major driver of morbidity, sometimes in combination with upstream type 2 pathway inhibition. Reported benefit has been described in STAT3 dominant-negative hyper-IgE syndrome where IL-5 pathway blockade reduced eosinophil burden and steroid dependence (see Table 2) (120). Similarly, a single case reported complete clinical remission in a patient with *STAT6* GOF disease by combining IL-4Rα and IL-5 inhibition, while mepolizumab alone was minimally effective (59).

### HSCT

In contrast to pathway-modulating therapies, HSCT aims to correct the underlying immune defect by replacing the whole hematopoietic compartment. HSCT plays an important but selective role in the management of PADs, reflecting the fact that PADs sit at the interface of immune dysregulation and immunodeficiency. At a biological level, HSCT is curative for PADs in which the dominant driver of disease is a cell-intrinsic hematopoietic defect. In these disorders, replacing the immune system corrects the underlying defect that drives severe allergy, immune dysregulation, and infection susceptibility.

Strong evidence for HSCT efficacy comes from disorders in which allergic disease coexists with combined immunodeficiency or profound immune dysregulation. HSCT is well established to be potentially curative in Omenn syndrome (most commonly caused by hypomorphic *RAG1* or *RAG2* variants) (121), WAS (122), IPEX (123), and DOCK8 deficiency (124). Historically, HSCT was not routinely recommended for AD-HIES (a.k.a. Job’s syndrome) because early experience suggested unpredictable benefits, particularly for nonimmune features such as skeletal and connective tissue abnormalities (71, 131). However, with improvements in transplant conditioning and supportive care, accumulating evidence indicates that HSCT can effectively correct the underlying immunological defects, reduce infection burden, and improve long-term outcomes in selected patients with DN-STAT3 deficiency, prompting a shift toward considering HSCT for severe phenotypes (7, 125, 126). These experiences have clarified the biological criteria that guide HSCT decision-making in PADs.

As a general rule, HSCT is appropriate in PADs only when the dominant pathology arises from hematopoietic immune cell failure that cannot be adequately controlled medically. When disease is driven primarily by epithelial barrier defects (such as Netherton syndrome due to *SPINK5* variants or ichthyosis vulgaris due to *FLG* variants), by GOF cytokine signaling (including *STAT6* or *JAK1* GOF disorders), or by partially preserved immune systems in which targeted biologic therapies are effective (e.g., CARD11-associated atopy), HSCT is typically not indicated. Nevertheless, the decision to pursue HSCT must be tailored to the individual patient after holistic consideration of the underlying disease mechanism, natural history of the specific disease, major clinical features, preexisting organ damage, and the available donor options. It should be noted that a number of the medications described above can also be effective when used in “bridge-to-transplant” therapeutic strategies with the goal to reduce inflammation before HSCT (132).

### Gene therapy

Advances in gene therapy are now shifting selected PADs from experimental intervention toward durable genetic correction. Gene therapy is emerging as a promising curative strategy for selected PADs caused by LOF variants in hematopoietic cells, particularly WAS, where lentiviral gene-corrected autologous hematopoietic stem cells have demonstrated stable engraftment, immune reconstitution, and sustained clinical improvement in infections, eczema, and autoimmune manifestations (133, 134, 135, 136, 137). Indeed, in December 2025, the U.S. Food and Drug Administration approved etuvetidigene autotemcel (Waskyra), a lentiviral gene therapy for WAS (138).

Collectively, these therapeutic advances illustrate how genetically defined PADs enable rational selection across a spectrum of interventions rooted in disease mechanism rather than phenotype alone.

## Challenges and opportunities

The study of PADs has advanced rapidly alongside expanding access to next-generation sequencing, yielding critical insights into type 2 immunity, barrier dysfunction, and immune regulation. However, translating these discoveries into consistent diagnosis and care remains challenging. Importantly, these challenges also highlight key opportunities for scientific discovery, clinical impact, and equitable translation.

### Increasing global clinical awareness of PADs

Despite increasing awareness among clinical immunologists and allergists, many PADs remain underdiagnosed, particularly when they present as severe or early-onset atopy without syndromic features (139). This “rare-in-common” paradigm was emphasized by a recent U.K. Biobank analysis in which 25 of 998 individuals (2.5%) diagnosed with atopic dermatitis carried pathogenic or likely pathogenic variants in 13 genes, including *FLG* (140, 141). Under-recognition is likely more pronounced in low- and middle-income countries, where limited access to genomic testing, clinical immunology expertise, and diagnostic infrastructure constrains detection. As a result, the global burden of PADs is skewed, with significant underreporting from under-resourced regions, likely missing both known and yet undiscovered PADs (1, 142). Addressing this gap is essential for health equity and for advancing PAD discovery, which depends on diverse population-scale observation.

### Complexity within monogenic disease

Contrary to early assumptions, not all PADs follow a straightforward Mendelian path (143). Variable expressivity, incomplete penetrance, and gene–environment interactions are increasingly recognized as modifiers of disease severity and presentation, even among individuals with identical variants. For example, *FLG* variants can lead to either mild eczema or severe ichthyosis vulgaris depending on the variant and environmental exposures such as microbiota, climate, and skin care practices (144). Likewise, patients with *STAT6* or *CARD11* variants may exhibit a spectrum of phenotypes based on variant type and host context (13, 14, 145, 146, 147). These observations reinforce the need for systems-level approaches that integrate multi-omics data, environmental exposures, and longitudinal clinical phenotyping to better define disease trajectories and therapeutic windows.

### Persistent therapeutic gaps

Although several monogenic PADs offer direct therapeutic targets such as dupilumab for *STAT6* GOF disease or JAKinibs for *JAK1* GOF disease, many disorders remain without effective or accessible treatment options. Conditions involving cytoskeletal defects or transcriptional regulators that lead to severe impairment of immune function or malignancy may still require HSCT, which is not feasible or accessible for all patients. Moreover, partial LOF variants or dominant-negative variants can complicate therapeutic targeting. These limitations highlight the need to expand therapeutic pipelines, including gene therapy, antisense oligonucleotides, and small-molecule or pathway-specific inhibitors that extend beyond canonical Th2 signaling.

### Bridging rare and common disease

One of the most exciting opportunities in PAD research lies in its potential to reshape how we understand common allergic diseases. Insights from monogenic PADs are helping define molecular endotypes within polygenic atopy. For example, population studies have identified common risk variants in *IL4RA*, *STAT6*, and *FLG* associated with atopic dermatitis and asthma, echoing mechanisms observed in monogenic forms (148, 149, 150, 151). This suggests a continuum between rare and common disease, where rare variants act as “natural experiments” that reveal core disease mechanisms. Going forward, integrating PAD insights into large-scale genotype–phenotype association studies and biobank analyses may help stratify patients with common atopy for more targeted interventions. It is now appreciated that monogenic human diseases substantially increase the probability of successful drug development because direct genetic perturbation of a single target provides causal validation of disease relevance, effect direction, and on-target safety. This principle is supported by large-scale analyses showing that drug targets with human genetic evidence are two- to threefold more likely to succeed in clinical development (152, 153).

## Looking ahead

PADs have moved beyond being rare diagnostic curiosities and now stand as powerful models for understanding human immunobiology and for advancing precision medicine. The field is now poised to move beyond gene discovery toward deeper biological understanding, improved clinical translation, and durable therapeutic innovation.

One of the most immediate challenges is genetic variant interpretation. As sequencing becomes routine, variants of uncertain significance in known PAD genes are increasingly encountered in clinical practice. Addressing this will require systematic functional validation using patient-derived cells, robust cellular assays, and shared experimental platforms that enable reproducible assessment of variant impact and bridge the gap between genetic discovery and clinically actionable interpretation. Equally important is ensuring that progress in PAD research is globally inclusive. Many affected individuals remain undiagnosed, particularly in regions with limited access to genomic testing and subspecialty care. Investment in infrastructure, training, and equitable access to sequencing will improve patient outcomes while accelerating discovery in genetically diverse populations. At the same time, PADs offer a bridge between rare disease research and common allergic conditions, as pathways revealed through monogenic disorders also operate in polygenic diseases such as asthma and atopic dermatitis.

Viewed collectively, PADs are a powerful example of how careful study of rare human disease can illuminate fundamental biology, guide precision therapy, and reshape our understanding of allergic inflammation. The next phase of the field lies not only in discovery, but also in integration. Our job is now to thoughtfully integrate biology with clinical care, rare disease with common disease, and innovation with equity.

## Data Availability

No new data were generated or analyzed in support of this review article.
